# Agonistic anti-CD40 promotes early development and increases the incidence of severe thyroid epithelial cell hyperplasia (TEC H/P) in CD4−/− mice

**DOI:** 10.1002/iid3.5

**Published:** 2013-10-30

**Authors:** Shiguang Yu, Edward F Downey, Helen Braley-Mullen

**Affiliations:** 1 Department of Veterans Affairs Research ServiceColumbia, Missouri, 65212; 2 Departments of Internal Medicine, University of Missouri School of MedicineColumbia, Missouri, 65212; 3 Molecular Microbiology and Immunology, University of Missouri School of MedicineColumbia, Missouri, 65212; 4 Department of Biological Science, Arkansas State University, Arkansas Biosciences InstituteJonesboro, Arkansas, 72467

**Keywords:** Autoimmunity, fibrosis, hyperplasia, inflammation, T cells

## Abstract

IFN-γ−/−NOD.H-2h4 mice develop thyroid epithelial cell hyperplasia (TEC H/P) characterised by abnormal proliferation of thyrocytes and infiltration of thyroids by CD4+ and CD8+ T cells, macrophages and dendritic cells. CD8+ T cells from mice with severe TEC H/P transfer similar lesions to SCID recipients, whereas CD4+ T cells transfer mild TEC H/P. CD4− and CD8− deficient IFN-γ−/−NOD.H-2h4 mice were generated to determine if CD4+ T cells were required for initial activation of the CD8+ T cells that transfer TEC H/P. After 6–8 months on NaI water, only 2 of 60 CD8−/− mice developed severe TEC H/P, whereas 31 of 101 CD4−/− mice developed severe TEC H/P and fibrosis comparable in severity to that of IFN-γ−/− mice. However, splenocytes from CD4−/− mice with severe TEC H/P did not effectively transfer severe TEC H/P to SCID recipients. When CD4−/− donors were given agonistic anti-CD40 mAb, most developed severe TEC H/P and their cells transferred severe TEC H/P to SCID recipients. These results indicate that agonistic anti-CD40 can provide an important signal for activation of autoreactive CD8+ T cells that transfer severe TEC H/P. Therefore, targeting or blocking CD40 could provide effective therapy for diseases involving hyperplasia and fibrosis mediated by CD8+ T cells.

## Introduction

IFN-γ−/− NOD.H-2h4 mice given NaI in their drinking water develop an autoimmune disease characterised by extensive proliferation of thyroid epithelial cells (TEC H/P) and development of thyroid fibrosis [1,2]. Thyroid hyperplasia in humans is very common and can be difficult to distinguish from neoplasia [3–5]. The animal model of thyrocyte hyperplasia in IFN-γ−/− NOD.H-2h4 mice is a well-characterised animal model that can be used to increase our understanding of the mechanisms underlying development of abnormal cell proliferation, hyperplasia and fibrosis in autoimmune disease.

Several autoimmune diseases such as systemic lupus erythematosus (SLE), systemic sclerosis, rheumatoid arthritis, and autoimmune thyroiditis can be associated with epithelial cell hyperplasia and fibrosis [2,6,7], and mechanisms underlying development of such lesions are poorly understood. Thyroid nodules and thyrocyte hyperplasia are very common in humans, and can be associated with an increased risk of thyroid cancer. Thyroid cancer is one of the most common endocrine tumours in humans and understanding the mechanisms that lead to dysregulated thyrocyte proliferation is very important [8,9]. TEC H/P in IFN-γ−/− NOD.H-2h4 mice is an autoimmune disease, because T cells from IFN-γ−/− mice with severe TEC H/P transfer similar lesions to IFN-γ−/− NOD.H-2h4.SCID mice, and IFN-γ−/− SCID mice, which lack lymphocytes, and mice lacking T cells do not develop TEC H/P [2,10]. Purified CD8+ T cells from IFN-γ−/− mice with severe TEC H/P transfer severe TEC H/P to SCID recipients, whereas purified CD4+ T cells transfer mild TEC H/P [10].

Our previous studies clearly showed that fully activated highly purified CD8+ T cells could transfer severe TEC H/P to SCID recipients [10]. However, the induction period for development of severe TEC H/P is long [2,10], and CD4+ T cells could be required for initial activation of the CD8+ effector T cells. CD4+ T cells play an important role in development of memory CD8+ T cells in several models [11–14], including diabetes in NOD mice [15]. In those studies, memory CD8+ T cells that developed in the absence of CD4+ T cell help had reduced survival and effector function compared to memory CD8+ T cells that developed in the presence of CD4 help.

CD4−/− and CD8−/− IFN-γ−/− NOD.H-2h4 mice were developed to determine if T cells that induce and transfer severe TEC H/P can develop in the absence of CD4+ T cells. The results showed that 30% of CD4−/− mice developed severe TEC H/P, whereas CD8−/− mice were almost completely resistant. These results demonstrate that CD4+ T cells are not absolutely required for development of severe TEC H/P, but splenocytes of CD4−/− mice were deficient in their ability to transfer severe TEC H/P to SCID recipients. When CD4−/− mice were given agonistic anti-CD40, most of them developed severe TEC H/P, and their splenocytes could transfer severe TEC H/P. These results suggest that a signal that can be provided by anti-CD40 is important for activation of T cells from CD4−/− mice to transfer severe TEC H/P.

## Materials and Methods

### Mice

IFN-γ−/− NOD.H-2h4 and IFN-γ−/− NOD.H-2h4 SCID mice were generated as previously described [1,2]. CD4−/− and CD8−/− IFN-γ−/− NOD.H-2h4 mice were generated by crossing CD4−/− or CD8−/− NOD males (Jackson Laboratories, Bar Harbor, ME, USA) with NOD.H-2h4 females. F1 mice were bred and F2 mice were selected for expression of the H-2K^k^ MHC class I molecule by flow cytometric analysis of peripheral blood [1] and for the CD4−/− or CD8−/− knockout by PCR analysis of tail DNA according to the protocols on the Jackson Laboratories web site. After selection of mice homozygous for H-2K^k^ and the gene knockout, CD4−/− and CD8−/− NOD.H-2h4 mice were further crossed with IFN-γ−/− NOD.H-2h4 mice to generate CD4−/− and CD8−/− IFN-γ−/− NOD.H-2h4 mice. All mice used in this study are NOD.H-2h4 and IFN-γ−/−; for simplicity, the NOD.H-2h4 and IFNγ−/− designations are not included in every instance.

Except where mentioned otherwise, all mice were given 0.08% NaI water for 6–8 months beginning at 7–8 weeks of age [1,2]. In some experiments, CD4−/− mice were given a single i.p. injection of 200 µg anti-CD40 FGK45 (BioXcell, West Lebanon, NH, USA) or IgG2a isotype control, and used at various intervals thereafter as indicated for individual experiments. For the experiments in Figures 3B and 6, anti-CD40 or isotype control was injected on the day the mice were given NaI water, while in the experiments in Figures 3A and 4, the mice were given anti-CD40 or isotype control 5–8 months after they started on NaI water (also see figure legends). 100–200 µg of anti-CD40 was determined in preliminary experiments to result in optimal stimulation of splenic APC and to effectively promote development of severe TEC H/P. All mice were given NaI in their drinking water at the time of injection of anti-CD40 or isotype control. All animal protocols were approved by the University of Missouri and VA Animal Care and Use Committees.

### Cell culture and adoptive transfer

Splenocytes from CD4−/− and CD4+ IFN-γ−/− NOD.H-2h4 mice with severe TEC H/P were pooled and cultured for 72 h with mouse thyroglobulin (MTg) as previously described [10]. In some experiments, IL-2 (10 ng/mL) or anti-CD40 (FGK45) (10 µg/mL) was added to cultures of CD4−/− splenocytes, and in other experiments, donor mice were given 200 µg anti-CD40 1–2 months prior to splenocyte culture. Splenocytes (3 × 10^6^) were transferred i.v. into NOD.H-2h4 IFN-γ−/− SCID mice, recipients were given NaI water, and thyroid histology was assessed 28 or 60 days later as indicated in the figures. The SCID recipients were 7–10 weeks old at the time of cell transfer. In most experiments, cultured cells were examined by flow cytometry prior to transfer to ensure absence of CD4+ T cells. Thirty to 40% of the transferred cells were CD8+ T cells and 40–50% were CD19+ B cells. In addition, spleens of SCID recipients were examined at the time thyroids were removed to ensure that recipients of CD4−/− splenocytes did not have any CD4+ T cells and that the SCID recipients were not ‘leaky’. As shown previously, splenocytes from IFN-γ−/− donors with severe TEC H/P transfer severe TEC H/P to SCID recipients, whereas splenocytes from donors with no or mild TEC/HP do not transfer severe TEC H/P [2,10]. The TEC H/P severity scores of donors used for all experiments were shown to be 4–5+ by histology prior to transferring their splenocytes. Culturing donor cells in vitro prior to transfer greatly facilitates the efficiency of cell transfer and allows transfer of severe TEC H/P with 10-fold fewer cells than are needed with freshly isolated splenocytes [10].

### Flow cytometry

Spleens of some experimental mice were analysed for expression of CD4, CD8, CD19, CD11c, CD40, CD80 and NKG2D by flow cytometry (FACScan and FACSCalibur) as previously described [10] by gating on total lymphocytes. Antibodies were obtained from eBioscience (San Diego, CA, USA) or Biolegend (San Diego, CA, USA).

### Evaluation of TEC H/P severity scores

Thyroids were removed at various times as indicated in the figures and table. One thyroid lobe was fixed in formalin, sectioned and stained with hematoxylin and eosin (H & E), and the other thyroid lobe was frozen for later use for analysis by IHC, Western blot or PCR as previously described [1,2]. All slides were scored blindly and independently by two individuals, and thyroid histopathology was scored for the extent of thyroid follicular cell hyperplasia/proliferation using a scale of 0–5+ as previously described [1,2]. Briefly, a score of 0 indicates a normal thyroid, and 0+ indicates mild follicular changes and/or a few inflammatory cells infiltrating the thyroids. A 1+ score is defined as hyperplastic changes sufficient to cause replacement of several follicles, 2+ represents hyperplastic changes causing replacement or destruction of up to 1/4 of the gland, 3+ indicates that 1/4–1/2 of the gland is destroyed by hyperplastic changes, and 4+ indicates that greater than 1/2 of the gland is destroyed. Thyroids given a score of 5+ had few or no remaining normal follicles and extensive collagen deposition (fibrosis). Severe TEC H/P lesions (graded 4–5+ based on the percentage of normal thyroid follicles remaining) were greatly enlarged and white in color, and had widespread clusters of proliferating thyrocytes and some lymphocyte infiltration. The areas of proliferating thyrocytes were usually surrounded by collagen. All thyroids with mild or severe hyperplasia had infiltrating mononuclear cells, consisting of T cells, macrophages and dendritic cells [10].

### Masson's trichrome

Fibrosis was evaluated using Masson's trichrome stained thyroid sections as previously described [16].

### Serum T4 assays

In some experiments, serum T4 levels were determined using T4 ELISA kits (Leinco, Inc., St. Louis, MO, USA). Values for normal mouse serum ranged from 4 to 8 µg/dL of serum and values were considered low when <3 µg/dL [2,17]. All mice with low serum T4 had very severe (4–5+) TEC H/P with few or no normal thyroid follicles, whereas all mice with severity scores <4+ or 4+ severity scores with significant numbers of remaining follicles have serum T4 levels in the normal range. Two samples of serum from normal mice are also included in each assay, and they are always within the range of 4–8 µg/dL.

### Immmunohistochemistry

Thyroid tissues were embedded in OCT (Sakura, Torrance, CA, USA), and sections (7 µm) were cut and stored at −80°C. Frozen thyroid sections were stained as previously described [1,2]. Anti-CD4 (GK1.5), anti-CD8α (53.6), anti-CD11b (CRL 1969, ATTC), and anti-CD11c (N418, Biolegend) were used as primary antibodies. Biotinylated goat-anti-rat IgG or biotinylated hamster IgG (Jackson Immunoresearch, West Grove, PA, USA) was used as secondary antibody, followed by incubation with Vectastain Elite avidin–biotin complex (Vector Laboratories, Burlingame, CA, USA). Peroxidase activity was visualized using Nova Red substrate (Vector) [2,18,19].

### Statistical analysis

The Wilcoxson rank sum test was used to analyse differences in disease severity scores between groups of mice. Student's *t*-test was used for all other analyses. Results are expressed as mean ± SEM. Values of *P* < 0.05 were considered significant.

## Results

### CD4−/− mice develop severe TEC H/P and CD8−/− mice are resistant to TEC H/P

Our previous studies showed that 60–70% of IFN-γ−/− NOD.H-2h4 mice given NaI in their drinking water for >6 months develop severe (4–5+) TEC H/P [1,2]. As stated in the Introduction, CD8+ T cells are the major effector cells for TEC H/P, and after CD8+ T cells are activated, CD4+ T cells are not required to transfer severe TEC H/P [10]. The induction period for development of severe TEC H/P is long (>6 months), and our earlier studies did not address whether CD4+ T cells might be required for the initial activation of CD8 cells and development of severe TEC H/P in IFN-γ−/− donors. CD4 and CD8-deficient IFN-γ−/− NOD.H-2h4 mice were developed in order to address this question. Mice were given NaI in their water for 6–8 months. The results (Table1) showed that 31 of 101 (31%) CD4−/− mice develop 4+ to 5+ severe TEC H/P, whereas most other CD4−/− mice developed no or very mild (0+ to 1+) TEC H/P. In contrast, most CD8−/− mice were resistant to TEC H/P, as only 2 of 62 (3%) CD8−/− mice developed severe (4–5+) TEC H/P after 7 months on NaI water. The reduced incidence of severe TEC H/P in CD4−/− mice is almost completely nullified if CD4−/− mice are given CD4+ T cells (splenocytes from CD8−/− donors), since the incidence of severe TEC H/P (55%; Table1, line 3) was comparable to that of IFN-γ−/− NOD.H-2h4 mice (60–70%) as shown in our earlier studies [1,2] and in Table1, line 4. These results indicate that CD8+ T cells can be activated to induce severe TEC H/P in the absence of CD4+ T cells, but the incidence of severe TEC H/P is much higher when CD4+ T cells are present.

**Table 1:** Development of severe TEC H/P in IFN-γ−/− CD4−/− mice

Mice^1^	0	1+	2+	3+	4+	5+
TEC H/P severity^2^						
CD8−/−	50	6	3	1	1	1
CD4−/−	63	3	3	1	7	24
CD8−/− to CD4−/−	6	0	1	1	0	9
IFN-γ−/−	6	0	0	0	1	15

1 IFN-γ−/− CD8−/− and CD4−/− NOD.H-2h4 mice, CD4−/− mice given splenocytes from CD8−/− NOD.H-2h4 mice and IFN-γ−/− NOD.H-2h4 mice were given NaI in their drinking water. Thyroids were removed 6–8 months later.

2 Numbers of mice with various degrees of severity of TEC H/P. Line 1 versus line 2, *P* < 0.01; line 2 versus line 3, *P* < 0.05.

**Figure 1:**
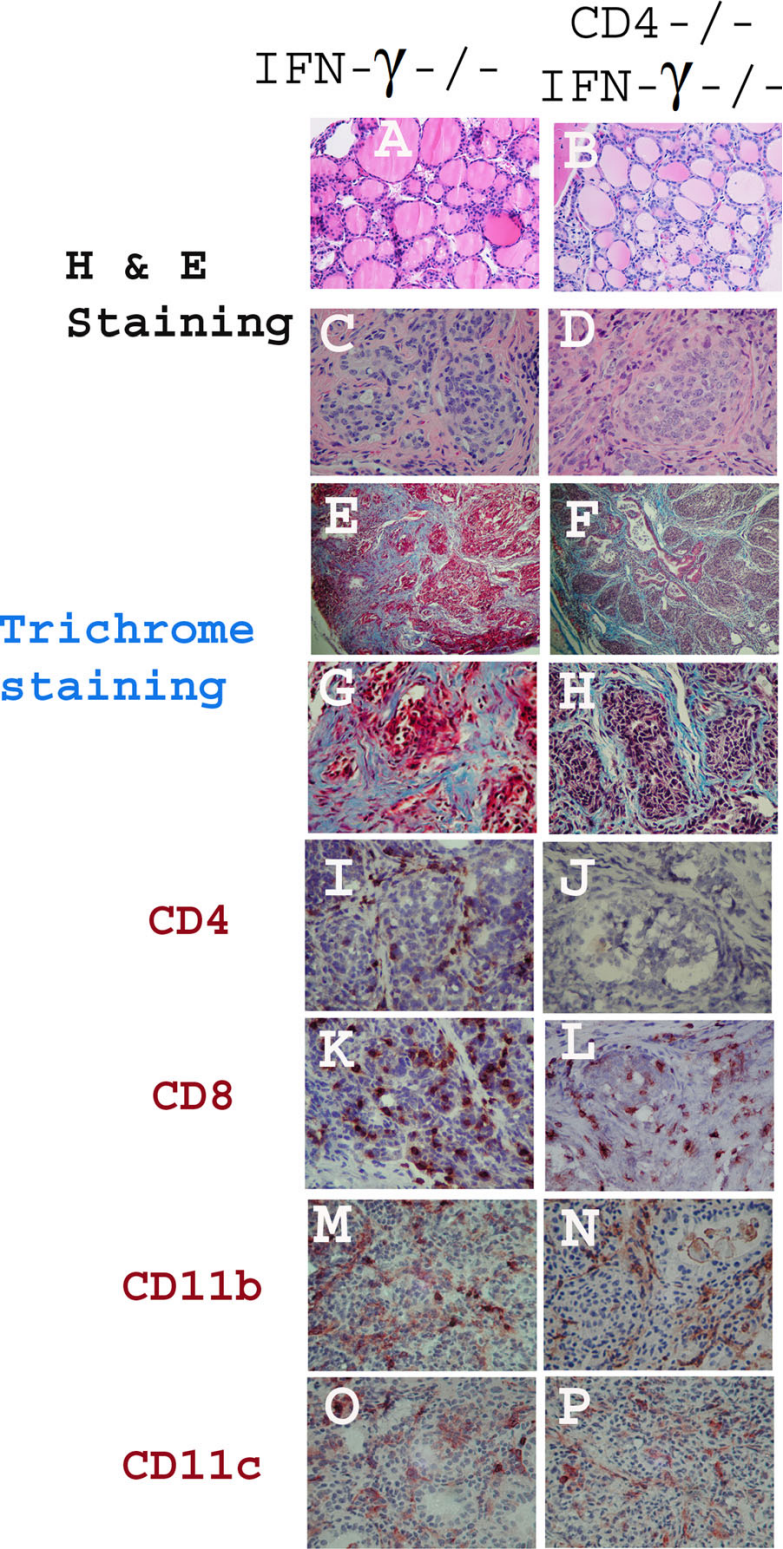
Histology of severe TEC H/P in CD4+ IFNγ−/− and CD4−/− IFNγ−/− mice. CD4−/− and CD4+ IFNγ−/− NOD. H-2h4 mice were given NaI in their drinking water, and thyroids were removed 6–7 months later. Shown are representative thyroid sections from naïve IFNγ−/− and CD4−/− mice (no TEC H/P) (A, B), and mice with severe (5+) TEC H/P (C, D) and fibrosis (E–H, blue colour). Thyroids of CD4−/− mice have many CD8+ T cells infiltrating the thyroid (L) and no CD4+ T cells (J) compared to CD4+ IFN-γ−/− mice that have both CD4+ and CD8+ T cells in thyroids (I, K). CD4+ IFN-γ−/− mice and CD4−/−IFNγ−/− mice have comparable numbers of CD11b+ (M, N) and CD11c+ (O, P) cells infiltrating their thyroids. H & E staining, A–D; Trichrome staining, E–H; IHC staining, I–P; Magnification: A–D, G–P, 400×; E, F, 100×. Results are representative of at least six different mice of each stain.

The histopathology of severe TEC H/P in CD4−/− IFN-γ−/− mice is like that of IFN-γ−/− mice, with extensive proliferation of thyrocytes (Fig. 1C and D) and collagen deposition (fibrosis, blue) surrounding the proliferating thyrocytes (Fig. 1E–H). IFN-γ−/− mice with severe TEC H/P always have both CD4+ and CD8+ T cells infiltrating their thyroids (Fig. 1I and K), whereas thyroids of CD4−/− mice have CD8+ T cells (Fig. 1L), but no CD4+ T cells (Fig. 1J). IFN-γ−/− mice and CD4−/− mice with severe TEC H/P have comparable infiltration of CD11b+ (Fig. 1M and N) and CD11c+ cells (Fig. 1O and P) in their thyroids.

### Splenocytes from CD4−/− mice with severe TEC H/P are deficient in their ability to transfer severe TEC H/P to SCID recipients

**Figure 2:**
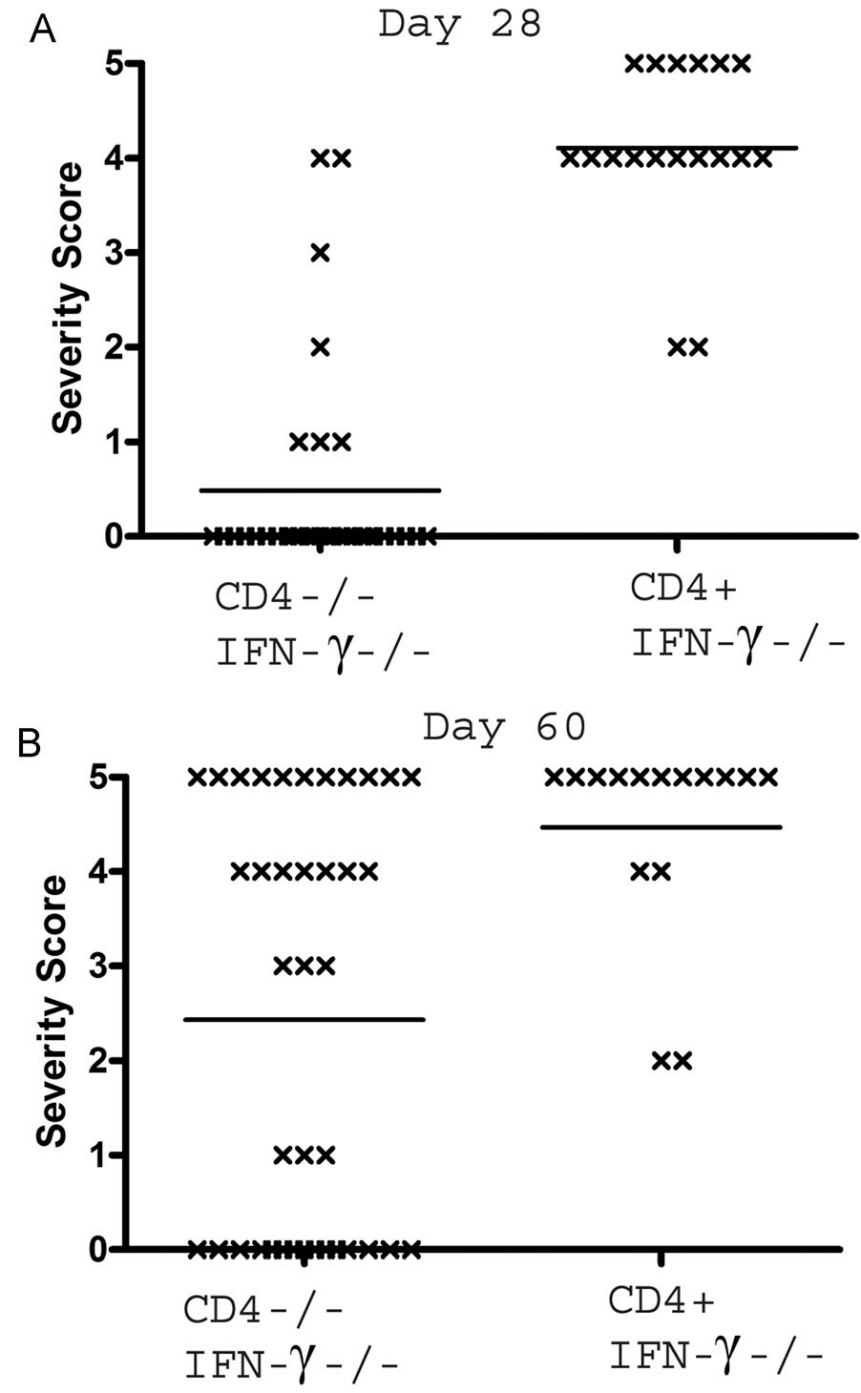
Splenocytes from CD4−/−IFNγ−/− donors with severe TEC H/P are deficient in their ability to transfer severe TEC H/P to SCID recipients. Splenocytes from CD4−/−IFNγ−/− and CD4+ IFN-γ−/− donors with severe (4–5+) TEC H/P were cultured for 72 h as described in Materials and Methods Section and transferred to SCID recipients. Thyroids were removed 28 or 60 days later to determine TEC H/P severity. Each symbol represents an individual mouse with the indicated TEC H/P severity score. Splenocytes from CD4−/−IFN-γ−/− donors transfer less severe TEC H/P compared to IFN-γ−/− CD4+ donors at both day 28 (*P* < 0.01) and day 60 (*P* < 0.05). The results are combined from three separate experiments and are representative of six experiments. For the CD4−/− group with severity scores of 0 at day 28, *n* = 26, severity score of 0 at day 60, *n* = 15 and for 5+ severity at day 60, *n* = 11. The numbers of mice in the other groups are evident from the X's.

Cultured splenocytes from IFN-γ−/− donors with severe TEC H/P or CD8+ T cells purified from cultured splenocytes transfer severe TEC H/P to SCID recipients, with severe TEC H/P developing in most recipients 28 days after cell transfer [10]. We hypothesized that splenocytes from CD4−/− donors with severe TEC H/P should also transfer severe TEC H/P to IFNγ−/− SCID recipients, thus providing a useful model for determining the mechanisms by which CD8+ T cells promote thyrocyte proliferation. To address this question, splenocytes from IFNγ−/− CD4−/− or IFN-γ−/−CD4+ mice with severe TEC H/P were cultured as described in Materials and Methods Section and cells were transferred to SCID recipients as previously described [10]. Unexpectedly, when thyroids were removed 28 days later, only 2 of 33 recipients of IFNγ−/− CD4−/− splenocytes had severe (4–5+) TEC H/P, whereas 16 of 18 recipients of IFN-γ−/− splenocytes had severe TEC H/P (Fig. 2A). Sixty days after cell transfer, 18 of 39 (44%) recipients of CD4−/− splenocytes had very severe (4–5+) TEC H/P compared to 13 of 15 (>80%) recipients of IFN-γ−/− splenocytes (Fig. 2B). When experiments were terminated, analysis of recipient spleens by flow cytometry indicated that recipients of splenocytes from CD4−/− donors had CD8+ T cells and no CD4+ T cells at both day 28 and day 60 (data not shown), confirming that the donors were CD4−/− and that the SCID recipients were not leaky and did not provide any CD4+ T cells. These results indicate that splenocytes from CD4−/− mice with severe TEC H/P are deficient in their ability to transfer severe TEC H/P to SCID recipients.

### Agonistic anti-CD40 promotes development of severe TEC H/P in CD4−/− IFN-γ−/− mice

The results in Table1 and Figure 2 suggest that CD4+ T cells are important for optimal activation of CD8+ effector T cells for TEC H/P. Memory CD8+ T cells can be reactivated to function as effector cells in other models, and the function of CD4+ T cells can be replaced by addition of IL-2 or agonistic anti-CD40 during in vitro activation [11–15,20–22]. To determine if IL-2 or agonistic anti-CD40 could activate splenocytes from CD4−/− donors to transfer severe TEC H/P, cells were cultured with MTg together with 10 ng/mL IL-2 or 10 µg/mL anti-CD40 prior to transfer to SCID recipients. Neither of these improved the ability of CD4−/− cells to transfer severe TEC H/P (Table2).

**Table 2:** Culturing splenocytes from CD4−/− mice in the presence of rIL-2 or anti-CD40 has no effect on their ability to transfer TEC H/P to SCID recipients

Mice^1^	Culture	0	1+	2+	3+	4+	5+
TEC H/P severity^2^							
CD4−/−	MTg	7	0	0	0	2	0
CD4−/−	MTg + IL-2	9	0	0	0	1	0
CD4−/−	MTg	6	0	0	0	0	0
CD4−/−	MTg + αCD40	6	1	0	1	0	0

1 CD4−/−IFNγ−/− NOD.H-2h4 mice were given NaI in their drinking water for 7–8 months. Splenocytes from donors with severe TEC H/P (4–5+ based on histology) were cultured with MTg alone or with MTg and 10 ng/mL IL-2 (line 2) or 10 µg/mL anti-CD40 (line 4). Cells were harvested and transferred i.v. (3 × 10^6^/recipient) to SCID recipients (see Materials and Methods Section).

2 Numbers of mice with the indicated TEC H/P severity scores 28 days after cell transfer.

**Figure 3:**
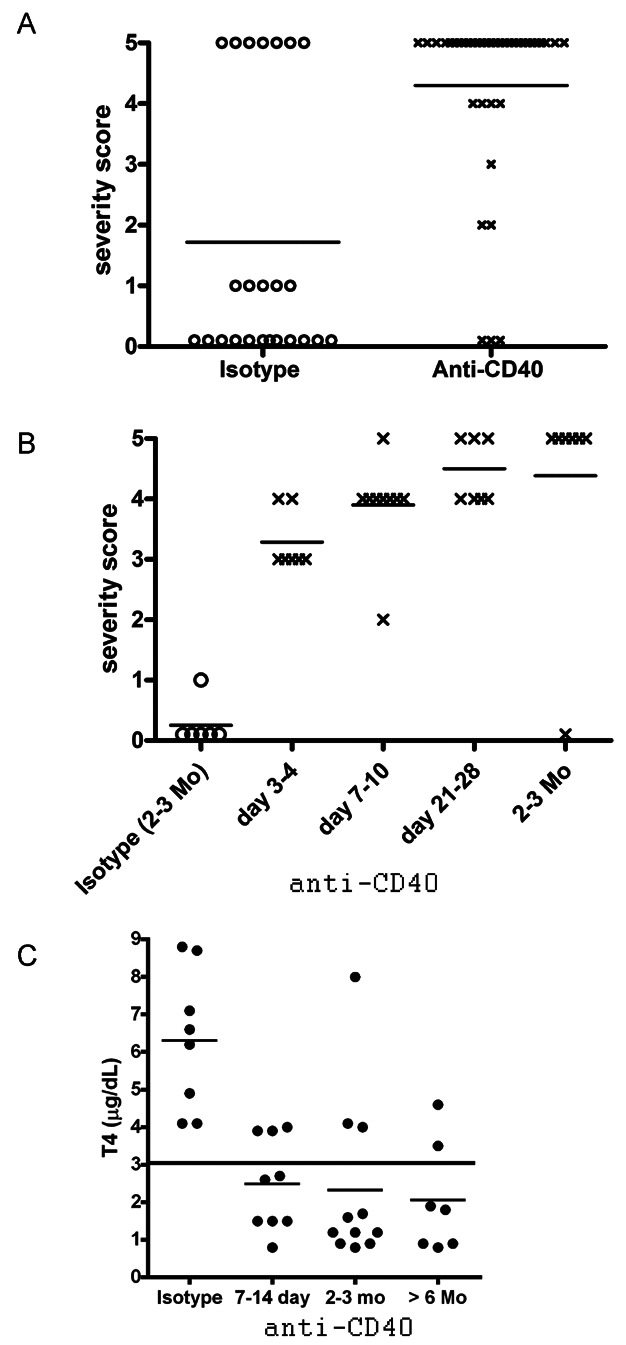
Agonistic anti-CD40 promotes earlier development and a greater incidence of severe TEC H/P in CD4−/− IFN-γ−/− NOD.H-2h4 mice. (A) CD4−/− IFNγ−/− donor mice, 7–8 weeks old, were given NaI in their drinking water for 6–8 months. Mice were given 200 µg anti-CD40 or isotype control 4–6 months later and TEC H/P severity scores were determined 4–8 weeks later (after 5–8 months on NaI water). (B) CD4−/− IFN-γ−/− NOD.H-2h4 mice were given 200 µg anti-CD40 or isotype control, on the same day they were given NaI in their drinking water. Thyroids were removed at the indicated times to determine TEC H/P severity. TEC H/P severity scores were significantly higher in all groups given anti-CD40 compared to those given isotype control. In A, 7 of 24 (28%) mice given isotype control and NaI water for >6 months had severe TEC H/P compared to 32 of 38 (>80%) age matched mice given anti-CD40 (*P* < 0.01). The group with 5+ severity scores after anti-CD40 consists of 24 mice. In B, none of the mice given isotype control had severe thyrocyte proliferation after 2–3 months on NaI water, while almost all mice had severe thyrocyte proliferation 7 days after injection of anti-CD40 (*P* < 0.01). (C) CD4−/− mice given agonistic anti-CD40 have low serum T4 when they have very severe TEC H/P. Each symbol represents the serum T4 level of an individual mouse. All mice with low serum T4 (<3 µg/dL as indicated by the line) had very severe 4–5+ TEC H/P, whereas mice with lower TEC H/P severity scores, including those scored 4+ but with significant remaining normal follicles, had normal serum T4. Mice in C include some, but not all, of the mice in B. Results shown in B are pooled from three different experiments and those in A are from seven different experiments.

The lower incidence of TEC H/P and reduced ability of cells from CD4−/− donors to transfer severe TEC H/P could be explained if CD8+ T cells were ineffectively primed in vivo in the absence of CD4+ T cells. One mechanism by which CD4+ T cells promote activation of CD8+ T cells is by activating and inducing maturation of APC such as B cells, dendritic cells and macrophages [21,23]. This function can often be replaced by agonistic anti-CD40 mAb [15,20,22]. We hypothesised that agonistic anti-CD40, by activating CD40+ APC, such as dendritic cells, macrophages or B cells, might facilitate activation of CD8+ T cells in CD4−/− mice [20–22], resulting in a greater incidence of severe TEC H/P. To test this hypothesis, CD4−/− mice were given NaI water. Some mice were given a single injection of anti-CD40 or isotype control 4–6 months later, and thyroids and spleens were removed 1–2 months later. Most (29 of 35) CD4−/− donors given anti-CD40 develop severe TEC H/P, whereas only 7 of 24 (30%) mice given isotype control developed severe TEC H/P (Fig. 3A). In addition to the increased incidence of severe TEC H/P, most mice had severe thyrocyte proliferation 4–7 days after injection of anti-CD40 (Fig. 3B). Indeed, nearly all CD4−/− mice given anti-CD40 had severe thyrocyte proliferation that developed quickly and persisted for several months after a single injection of anti-CD40 (Fig. 3B). Consistent with our earlier results, mice given isotype control did not develop TEC H/P (Fig. 3B) unless they were given NaI water for >6 months as in Figure 3A. These results indicate that agonistic anti-CD40 provides a signal that results in activation of CD8+ T cells in CD4−/− mice so that a high incidence of severe TEC H/P develops in the absence of CD4+ T cells. Importantly, most mice given agonistic anti-CD40 had low serum T4 levels, that is, they were clinically hypothyroid (Fig. 3C). Serum T4 levels always correlated with TEC H/P severity scores. All mice with 5+ severity scores or 4+ severity scores with few normal thyroid follicles had low serum T4 levels, while those with significant numbers of residual normal thyroid follicles (including most with 4+ severity scores) had normal serum T4 levels (Fig. 3C).

### Splenocytes from CD4−/− mice given anti-CD40 transfer severe TEC H/P to SCID recipients

**Figure 4:**
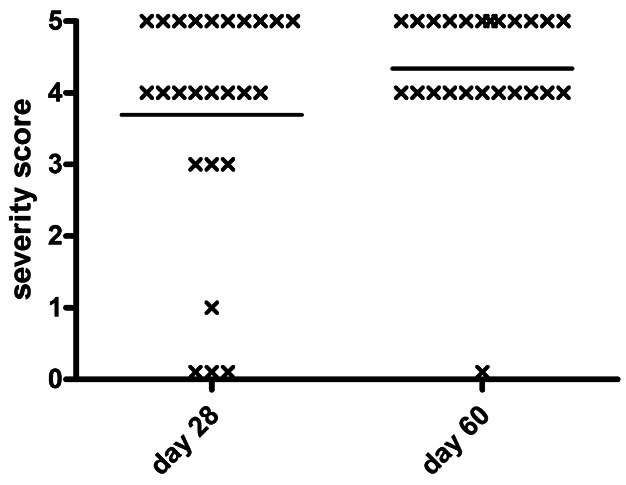
Splenocytes from CD4−/− IFNγ−/− mice given agonistic anti-CD40 transfer severe TEC H/P to SCID recipients. Splenocytes from CD4−/− IFNγ−/− donors with 4–5+ TEC H/P severity scores given anti-CD40 as in Figure 3A were cultured as described in Materials and Methods Section and transferred to SCID recipients. Thyroids were removed 28 or 60 days later to determine TEC H/P severity. Most recipient mice developed severe (4–5+) TEC H/P 28 or 60 days later, in contrast to recipients of splenocytes from age-matched CD4−/− IFNγ−/− donors not given anti-CD40 shown in Figure 2. Results are pooled from three separate experiments and are representative of at least five experiments.

To determine if anti-CD40 promoted activation of CD8+T cells able to transfer severe TEC H/P to SCID recipients, splenocytes from CD4−/− donors given anti-CD40 were cultured as described in Materials and Methods Section and transferred to SCID recipients. Recipients were given NaI water, and thyroids were removed 28 or 60 days later (Fig. 4). Splenocytes from donors given anti-CD40 transferred severe TEC H/P to 18 of 25 (72%) SCID recipients after 28 days, and 60 days after cell transfer, 23 of 24 (96%) recipients had severe TEC H/P (Fig. 4). These results are in contrast to those in Figure 2 where splenocytes from donors that were not given anti-CD40 did not transfer severe TEC H/P to the majority of recipients even though both groups of donors had comparable 4–5+ TEC H/P severity scores. All mice with 5+ severity scores at day 28 or day 60 and most with 4+ severity scores at day 60 had low serum T4, whereas most mice with 4+ severity scores at day 28 and all mice with severity scores of <4 had normal serum T4 (data not shown).

**Figure 5:**
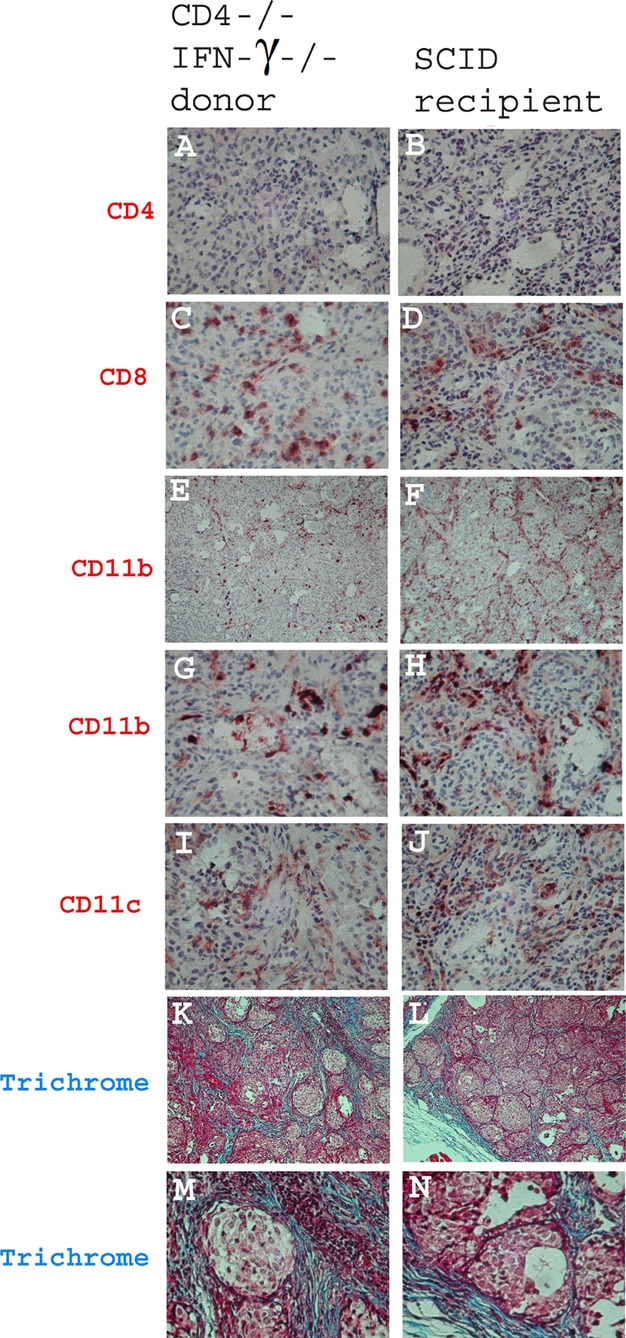
Inflammatory cells in thyroids of CD4−/− IFNγ−/− donors given anti-CD40 and SCID recipients of splenocytes from CD4−/− IFNγ−/− donors given anti-CD40. CD4−/− IFN-γ−/− mice were given anti-CD40 or isotype control as in Figure 3A. Splenocytes from donors with severe TEC H/P were cultured and transferred to SCID recipients, and thyroids were removed 28 (A–J) or 60 (K–N) days later. Both donor (A, C, E, G, I) and recipient mice (B, D, F, H, J) have similar inflammatory cell infiltration of thyroids. There were no CD4+ T cells (A, B), and many CD8+ T cells (C, D) in thyroids. CD11b+ (E–H), and CD11c+ cells (I, J) were also detected in both donor and recipient thyroids. Thyroids of SCID recipients of donor CD4−/− IFNγ−/− mice given anti-CD40 have severe fibrosis 60 days after cell transfer (L, N) comparable to the CD4−/− IFNγ−/− donors (K, M). Magnification: A–D, G–J, M, N, 400×; E, F, K, L, 100×.

CD4−/− mice given anti-CD40 had severe TEC H/P with infiltration of thyroids by CD8+ T cells, CD11b+ cells and CD11c+ cells, and no detectable CD4+ T cells (Fig. 5A, C, E, G and I). Histologically, these thyroids were indistinguishable from those of CD4−/− mice that develop severe TEC H/P without injection of anti-CD40 (Fig. 1). TEC H/P in SCID recipients of splenocytes from CD4−/− mice given anti-CD40 was indistinguishable histologically from that of the CD4−/− donors (Fig. 5B, D, F, H and J), indicating that severe TEC H/P can develop in recipients that lack CD4 cells. Thyroids of SCID recipients of splenocytes from CD4−/− donors given anti-CD40 had severe fibrosis 60 days after cell transfer comparable to that in donors (Fig. 5K–N).

### Agonistic anti-CD40 induces changes in splenic APC and splenic CD8+ T cells

**Figure 6:**
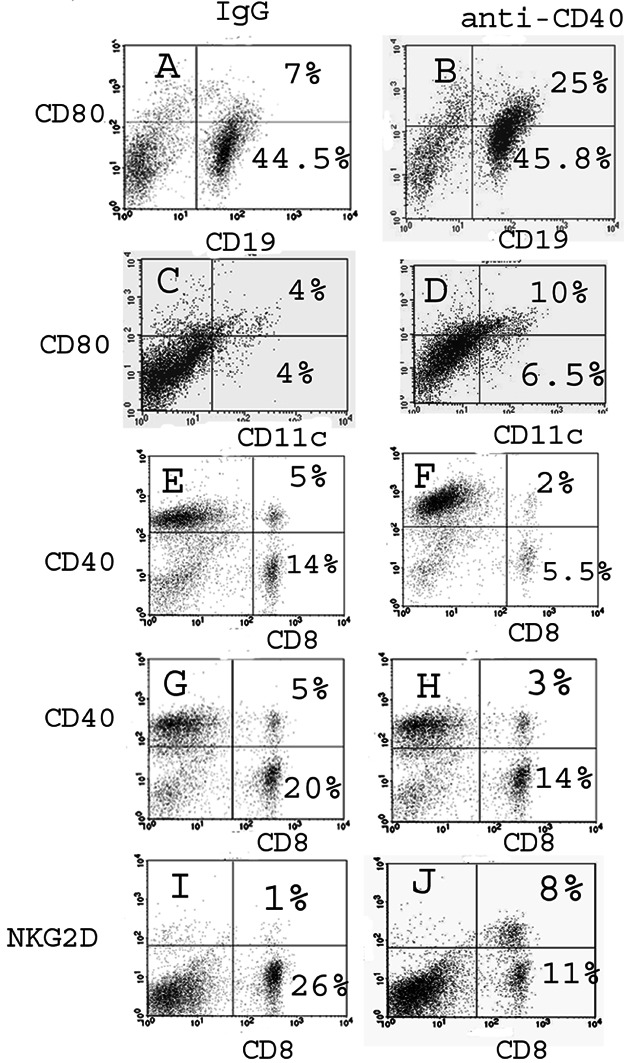
CD19+ B cells and CD11c+ cells are increased in spleens of CD4−/− mice given anti-CD40, and both cell populations have increased expression of CD80. CD8+ T cells in mice given anti-CD40 have increased expression of NKG2D. CD4−/− mice were given anti-CD40 (200 µg) or isotype control. Splenocytes were obtained 4 (A–F) and 10 (G–J) days later, and analysed by flow cytometry. Shown are representative flow cytometry results. Compared to mice given isotype control, B cells and CD11c+ cells in mice given anti-CD40 had increased expression of CD80 (B vs. A, D vs. C) at day 4 and increased expression of NKG2D on CD8 T cells at day 10 (J vs. I). Anti-CD40 had no effect on CD40 expression of CD8+ T cells compared to isotype control (G–J). Note that the percentage of CD8+ T cells is reduced in spleens of mice given anti-CD40 due to the increased percentage of B cells and increased numbers of spleen cells. Results are representative of 6–8 mice at each time point. The spleens from the mice used for analysis of B cells and CD11c+ cells 4 days after injection of anti-CD40 had 8.8 × 10^7^ cells (isotype control) versus 17.7 × 10^7^ cells (anti-CD40). For analysis of T cells 10 days after injection, the control mouse had 9.2 × 10^7^ cells/spleen while the anti-CD40 treated mouse had 12.8 × 10^7^ cells per spleen.

Since anti-CD40 is thought to promote activation of CD8+ T cells primarily by activating APC, it was important to determine the effects of anti-CD40 on splenic APC and T cells in CD4−/− IFN-γ−/− NOD.H-2h4 mice. Anti-CD40 induced early increases in both the percentages and numbers of splenic B cells and CD11c+ cells (Fig. 6), and both populations of APC expressed higher levels of B7.1 compared to B cells and CD11c+ cells of mice given isotype control (Fig. 6). Spleens of mice given anti-CD40 had nearly twice as many cells compared to mice given isotype control 3–7 days after injection of anti-CD40, but by day10–14, mice given anti-CD40 had 20–30% more spleen cells than mice given isotype control (data not shown and Fig. 6 legend). NKG2D expression increases on CD8+ T cells after activation [24]. NKG2D is higher on CD8+ T cells of mice with TEC H/P compared to CD8+ T cells in naive mice (unpublished results), and NKG2D expression on CD8+ T cells increased following injection of anti-CD40 (Fig. 6). Increased percentages and numbers of splenic APC were most evident 3–5 days following anti-CD40, whereas increases in activated T cells were not evident until day 10 or later (data not shown). These results suggest that agonistic anti-CD40 promotes activation of T cells able to transfer severe TEC H/P to SCID recipients, at least in part, through its ability to activate APC which then promote activation of CD8+ T cells able to transfer severe TEC H/P.

Taken together, these results indicate that CD8+T cells can be activated to induce severe TEC H/P in the absence of CD4+ T cell help, but administration of agonistic anti-CD40 leads to much earlier development and a greater incidence of severe TEC H/P. T cells from CD4−/− mice given agonistic anti-CD40 are more activated, since they can transfer severe TEC H/P to SCID recipients with a much higher efficiency compared to T cells from CD4−/− mice given isotype control.

## Discussion

CD4+ T cells play an important role in activation of CD8+ effector cells for immune responses against tumours and viruses, and also in autoimmune inflammation. In particular, CD4+ T cells are important to optimally activate CD8+ memory T cells to become effector cells [11–13,15,20–22]. In this study, CD4−/− and CD8−/− mice were developed to determine if CD4+ T cells were required for initial activation of CD8+ T cells, the major effectors for severe TEC H/P [10]. The results showed that CD4−/− mice develop TEC H/P comparable in severity to that of IFN-γ−/− mice but with a greatly reduced incidence, whereas CD8−/− mice are resistant to development of TEC H/P. Therefore, CD4+ T cells are not absolutely required for initial activation of the CD8+ T cells that induce severe TEC H/P, but the incidence of TEC H/P is much greater when CD4+ T cells are available. Moreover, the ability of splenocytes to transfer severe TEC H/P to SCID recipients was greatly compromised in the absence of CD4+ T cells (Fig. 2), suggesting that a signal provided by CD4+ T cells is important for activating CD8+ T cells that can transfer severe TEC H/P to SCID recipients.

CD4+ T cells provide help to both B cells and CD8+ T cells [25–27]. CD4+ T cells generally provide help to CD8+ T cells indirectly through their ability to activate APCs, for example, via CD40L/CD40 interactions [15,20,27–33]. CD40 is a TNF receptor family member expressed on APC, such as B cells, macrophages, and dendritic cells, as well as some T cells [34,35] and non-immune cells [29,30]. In the absence of CD4+ T cells, activation of CD8+ T cells by weakly immunogenic antigens or some autoantigens results in tolerance and/or ineffective development of memory cells [29,36]. The function of CD4+ T cells can sometimes be replaced by agonistic anti-CD40 which can promote CD8 T cell responses by activating APC [15,30–33], thereby promoting development of CD8+ memory and preventing induction of tolerance [30,36–39]. In this study, the incidence of severe TEC H/P in CD4−/− mice was greatly increased when mice were given agonistic anti-CD40 (Fig. 3). In addition, most mice had severe thyrocyte proliferation 7–10 days after injection of anti-CD40 (Fig. 3) compared to only 30% in IFN-γ−/− CD4−/− donors given NaI water for 7 months (Table1). T cells of CD4−/− mice given anti-CD40 also had a greatly improved ability to transfer severe TEC H/P to SCID recipients (Fig. 4). Because splenocytes from CD4−/− mice not given agonistic anti-CD40 did not effectively transfer severe TEC H/P to SCID mice (Fig. 2), T cells that develop following prolonged (>6 months) chronic stimulation in donor mice were apparently not fully activated in the absence of CD4+ T cells. Agonistic anti-CD40 provided a signal that activated these cells sufficiently so they could transfer TEC H/P to SCID recipients (Fig. 4). It is likely at least one important signal provided by agonistic anti-CD40 is to cross-link CD40, resulting in expansion and activation of splenic APC including B cells and CD11c+ cells with increased expression of costimulatory molecules such as B7.1 (Fig. 6). The activated APC promoted more effective activation of CD8+ T cells, as evidenced by their increased expression of NKG2D (Fig. 6). These results are consistent with other recent studies in which CD8+ memory cells activated during chronic infections or by persistent antigen in the absence of CD4 help (‘helpless CD8 cells’) required help from CD4+ T cells or a signal provided by anti-CD40 or CD40L in order to be reactivated to become effector cells [12–15,32].

In addition to the ability of anti-CD40 to induce activation of APC and promote activation of T cells able to induce severe TEC H/P in CD4−/− mice, anti-CD40 directly targets thyroid epithelial cells (TEC) of NOD and NOD.H-2h4 mice, leading to greatly increased expression of CD40, extensive thyrocyte proliferation, and production of proinflammatory cytokines in the thyroid [40]. The direct targeting of TEC by anti-CD40 is likely to be a major mechanism for the increased incidence of severe thyrocyte proliferation seen early after injection of anti-CD40 as shown in Fig. 3B. However, the ability of agonistic anti-CD40 to promote activation of splenocytes that transfer severe TEC H/P is not likely to be explained by direct effects of anti-CD40 on TEC, and is therefore more likely to be due to the ability of anti-CD40 to promote activation of splenic APCs and CD8 cells (Fig. 6). However, the anti-CD40 induced activation of splenic T cells that can transfer severe TEC H/P could be indirectly influenced by the effects of anti-CD40 on thyrocytes, since anti-CD40 interacts with CD40 on thyrocytes and promotes proliferation resulting in thyrocyte damage and loss of colloid in thyroid follicles [40]. Our working hypothesis is that anti-CD40 damages the thyroid, resulting in release of a thyroid antigen that can then be presented by anti-CD40-activated APC to activate CD8+ T cells in peripheral lymphoid organs. Experiments are currently in progress to address this important question.

The results of this study provide evidence for the importance of CD4+ T cells for activation of CD8+ T cells that function as effector cells for a chronic autoimmune disease of the thyroid, and show that a signal usually provided by CD4+ T cells can be provided by agonistic anti-CD40 when CD4+ T cells are absent. Therefore, blocking CD40-CD40L interactions could provide a means to treat autoimmune diseases involving CD8+ T cell-mediated epithelial cell hyperplasia and fibrosis in the thyroid as well as other tissues or organs [16,39,41,42]. Our results also suggest that agonistic anti-CD40 antibodies should be used with caution, since they can promote autoimmunity, particularly if a target organ such as the thyroid expresses CD40 [43,44].
